# The impact of a pandemic as an example of a stressful event, on anxiety and related emotional disorders of NF1 patients compared to healthy children

**DOI:** 10.3389/fpsyt.2025.1581465

**Published:** 2025-10-24

**Authors:** Rony Cohen, Sharon Aharoni, Ayelet Halevy, Jacob Genizi

**Affiliations:** ^1^ Department of Pediatric Neurology, Schneider Children’s Medical Center of Israel, Petah Tikva, Israel; ^2^ Neurofibromatosis Type 1 and Other Neurocutaneous Disorders Clinic, Schneider Children’s Medical Center of Israel, Petah Tikva, Israel; ^3^ Faculty of Medicine, Tel Aviv University, Tel Aviv, Israel; ^4^ Pediatric Neurology Unit, Bnai Zion Medical Center, Haifa, Israel

**Keywords:** neurofibromatosis type 1 (NF1), anxiety, COVID-19, separation anxiety disorder, social phobia

## Abstract

**Introduction:**

The COVID-19 pandemic has significantly affected global mental health, with children being particularly vulnerable. This study examines the psychological repercussions of the pandemic by comparing the prevalence of anxiety in healthy children and in those with Neurofibromatosis Type 1 (NF1), a hereditary disorder characterized by diverse clinical manifestations, including cognitive and behavioral challenges. The uncertainties of the pandemic may have influenced anxiety levels differently in these two populations.

**Results:**

A cross-sectional survey of 52 parents revealed that, although not statistically significant, more children with NF1 reported generalized anxiety and social phobia compared with their healthy peers. Conversely, healthy children tended to report more symptoms of somatization disorder, although this difference was not significant. Parent-reported scores showed a significant association between NF1 and separation anxiety disorder.

**Discussion:**

The findings suggest that the pandemic may have exacerbated pre-existing emotional challenges in children with NF1, potentially due to disruptions in healthcare access and increased social isolation. In contrast, typically developing children may have experienced stressors related to remote learning and social distancing. These results underscore the importance of tailored interventions and support for children with NF1 to address their specific emotional needs during crises.

**Conclusion:**

This study highlights the distinct psychological impact of the COVID-19 pandemic on typically developing children and those with NF1. While further research is needed to clarify the long-term effects, the findings emphasize the necessity of early screening and targeted interventions to mitigate emotional distress in children with NF1 during times of crisis.

## Highlights

- Children with NF1 exhibited trends toward generalized anxiety and social phobia during the pandemic.
- Healthy children, in contrast, tended to exhibit more symptoms of somatization disorder during the pandemic.
- The study underscores the importance of tailored interventions and support for children with NF1 to address their unique emotional needs during stressful events.

1. The small sample size is appropriate for preliminary exploration but may limit the generalizability of the results.
2. The use of questionnaires may limit response options, preventing participants from fully expressing nuanced or complex opinions.

## Introduction

Children with chronic diseases are reported to be at an increased risk of developing psychological and psychiatric disorders (1). Their ability to cope with stressful life events often differs from that of healthy peers, with posttraumatic stress symptoms notably more prevalent among children with chronic physical illnesses (2). The COVID-19 pandemic, a prolonged and unprecedented stressful event, has posed significant challenges to global health systems and profoundly affected mental health across populations (3). While the general population has experienced elevated stress and anxiety, it is important to examine how children and individuals with pre-existing medical conditions are uniquely affected (4).Neurofibromatosis Type 1 (NF1) is a hereditary disorder caused by mutations in the NF1 gene on chromosome 17q11.2, with a reported birth frequency ranging from 1 in 2,500 to 1 in 3,500 (5, 6). NF1 is characterized by multiple café-au-lait macules, skinfold freckling, Lisch nodules, and tumors affecting the nervous system, skin, and other organs (7). Additionally, children with NF1 frequently experience learning difficulties, attention-deficit hyperactivity disorder (ADHD), autism spectrum disorders (ASD), behavioral abnormalities, and psychosocial challenges, including anxiety (8).The COVID-19 pandemic has introduced stressors that significantly impact mental health, and children are no exception. Some studies report that individuals with chronic diseases experience higher levels of stress and anxiety than those without chronic illnesses (9–12). In contrast, other research does not support the hypothesis that having a chronic illness is associated with increased anxiety during the pandemic (13).This study aims to examine the psychological impact of the COVID-19 pandemic, focusing on anxiety in children with NF1 as an example of a stressful life event. By comparing anxiety prevalence in children with NF1 and their typically developing peers, this research seeks to clarify how pre-existing medical conditions may influence emotional responses during periods of crisis.

## Methods

### Study design

This study utilized a cross-sectional design to evaluate the psychological impact of the COVID-19 pandemic on children with NF1 compared to healthy peers.

### Participants

The study included children and adolescents aged 8 to 18 years, comprising two groups: healthy participants and patients with NF1. All NF1 patients were followed at a multidisciplinary NF1 clinic at Schneider Children’s Medical Center of Israel, a tertiary university-affiliated pediatric medical center. The diagnosis of NF1 was confirmed by a senior pediatric neurologist based on the 2021 National Institutes of Health criteria or genetic testing. Participants were recruited consecutively, and parents provided informed consent before participation.

### Ethical approval

The study protocol was reviewed and approved by the local ethics committee. Parental consent and assent from participants were obtained in accordance with ethical guidelines.

### Data collection

Data were collected through an anonymous, online cross-sectional survey completed by participants’ parents between September 2021 and September 2022. The survey included demographic data and validated psychological assessment tools.

### Anxiety symptoms assessment

The psychological impact was measured using the Screen for Child Anxiety-Related Emotional Disorders (SCARED, parent-reported Cronbach’s α = 0.85) (14),, a validated tool for screening anxiety symptoms in children and adolescents. The SCARED evaluates symptoms across several domains, including generalized anxiety disorder, separation anxiety disorder, panic disorder/somatization, social phobia, and school phobia. It is a reliable instrument for both child and parent reporting, consisting of 41 items and aligned with DSM-IV criteria for anxiety disorders.

### Statistical analysis

Data were analyzed using SPSS Statistics (version 27, IBM Corp). Fisher’s Exact Test was used to examine the association between categorical variables. The Mann–Whitney U test was applied to compare the distributions of ordinal variables (such as Likert scale responses) between the two groups. A p-value of < 0.05 was considered statistically significant.

## Results

**Figure 1:**
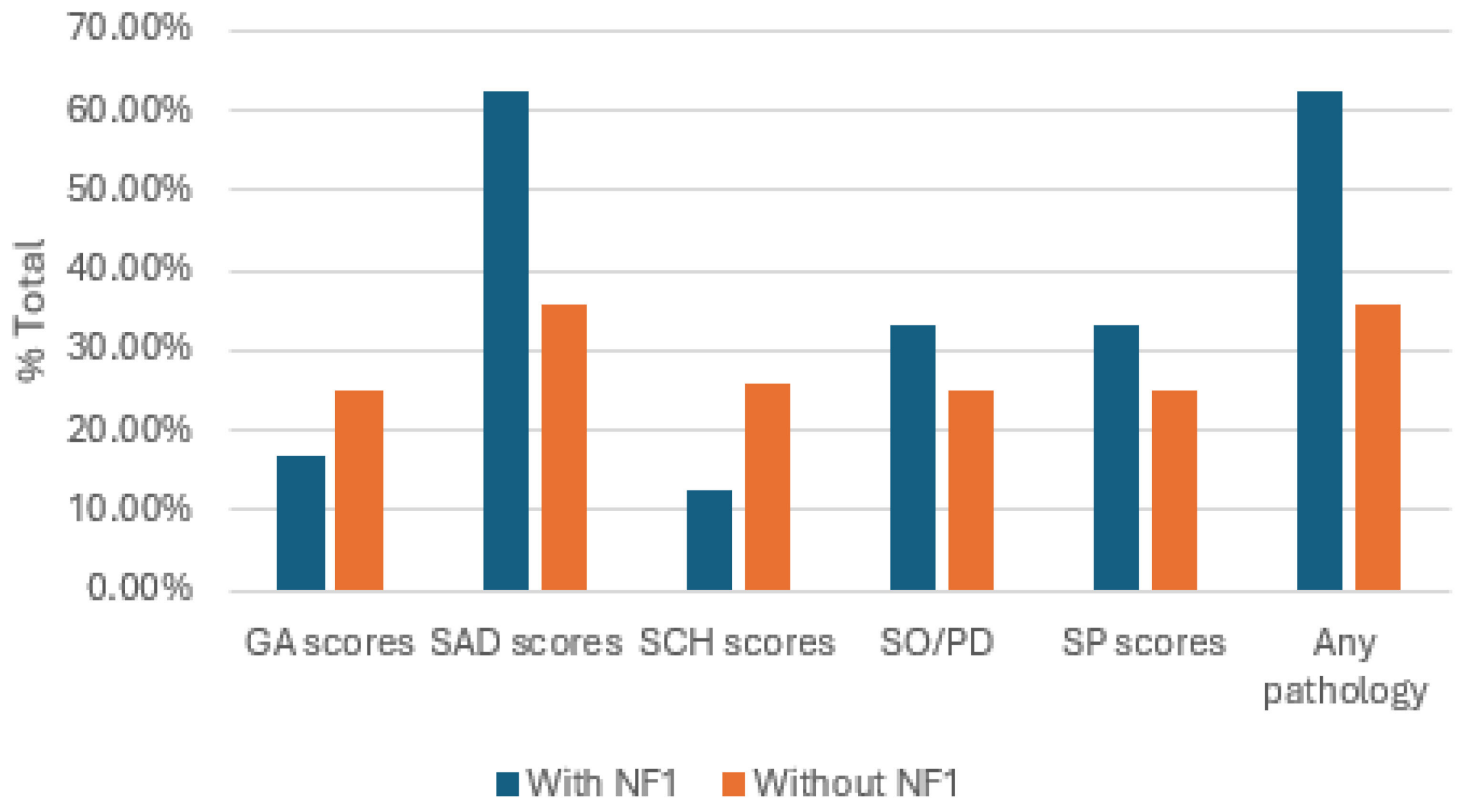
The distribution of SCARED scores among children with or without NF1. GA, general anxiety disorder; SAD, separation anxiety disorder; SO/PD, somatization /panic disorder; SP, social phobia; SCH, significant school avoidances.

A total of 52 parents participated in the e-survey regarding their children, with 28 reporting on children diagnosed with Neurofibromatosis Type 1 (NF1) and 24 on healthy children. Among the parents of children with NF1, only five also had a diagnosis of NF1 themselves. **Figure 1** illustrates the distribution of SCARED scores among the children. All psychological outcomes were reported by the parents regarding their children. Parent-reported data indicated that children with NF1 had a higher prevalence of certain anxiety-related symptoms compared to their healthy peers. Specifically, according to parent reports, children with NF1 were more likely to exhibit somatization symptoms (62.5%), social phobia (33%), and separation anxiety (33%). In contrast, parents of children without NF1 reported generalized anxiety symptoms more frequently, with 25% showing elevated levels. Parent-reported scores highlighted significant differences between the two groups (**Table 1**). Parents of children with NF1 reported markedly higher levels of separation anxiety symptoms (p < 0.05) and overall pathological anxiety symptoms (p < 0.05) compared to parents of healthy children. These differences were observed regardless of whether the reporting parent also had NF1, indicating no significant correlation between parent NF1 status and the symptoms reported in their children.

**Table 1:** SCARED Parent-report scores.

	With	NF1	Without	NF1	P value
	Normal Count/total%	Pathology Count/total%	Normal Count/total%	Pathology Count/total%	
GA scores cut off>9	20(83.3%)	4(16.7%)	21(75%)	7(25%)	P=0.85
SAD scores cut off >5	9(37.5%)	15(62.5%)	18(64.7%)	10(35.7%)	P=0.049*
SCH scores cut off >5	21(87.5%)	3(12.5%)	24(14.3%)	4(25.7%)	P=0.720
SO/PD cores cut off>7	16(66.6%)	8(33%)	21(75%)	7(25%)	P=0.360
SP scores cut off >8	20(66.6%)	4(33%)	22(75%)	6(25%)	P=0.783
Any pathology	9(37.5%)	15(62.5%)	18(64.7%)	10(35.7%)	P=0.049*

GA, general anxiety disorder; SAD, separation anxiety didorder; SO/PD, somatization/panic disorder; SP, social phobia; SCH, significant school avoidances.

## Discussion

Our study focused on children with NF1 and their anxiety during the COVID-19 pandemic, as reported by their parents. According to parent-reported data, children with NF1 were at an increased risk of specific anxiety-related symptoms (p < 0.05), particularly somatization, social phobia, and separation anxiety, compared to their healthy peers. In contrast, parents of children without NF1 reported that generalized anxiety appeared to be more common in their children. The observed differences in anxiety symptoms between children with NF1 and healthy children, as reported by their parents, can be attributed to several unique factors related to the biological, psychological, and social challenges of living with NF1 (7, 15). Children with chronic illnesses are more vulnerable to psychological distress, including anxiety and post-traumatic stress symptoms, due to ongoing medical and social challenges (16). NF1, in particular, is closely linked with neurodevelopmental disorders such as ADHD and ASD (15), which are known to heighten vulnerability to anxiety and social difficulties (17, 18). The COVID-19 pandemic, as an unprecedented global stressor, may have further amplified these risks. Interestingly, while our study found increased anxiety symptoms in NF1 children, prior research presents mixed findings. Some studies report that individuals with chronic diseases, including NF1, experience heightened anxiety due to social isolation and medical uncertainty during the pandemic (20). However, other studies suggest that the impact of COVID-19 on anxiety levels may vary depending on individual family environments, coping mechanisms, and access to psychological support systems (19). The increased prevalence of separation anxiety, social phobia, and somatization symptoms in NF1 children can be attributed to both genetic and environmental factors. NF1 is well known for its association with cognitive and behavioral impairments, particularly ADHD and ASD (15,). These underlying neurodevelopmental conditions may make NF1 children more susceptible to anxiety, particularly in response to stressful events such as the pandemic (7, 19).

Additionally, the impact of social isolation, disruptions in routine, and increased medical vulnerabilities during COVID-19 could have amplified anxiety symptoms in this population (22). Studies indicate that NF1 children are at a higher risk for social difficulties due to deficits in executive function, emotional regulation, and peer relationships (23). Separation anxiety and social phobia are commonly reported in NF1 children, possibly due to difficulties in processing social cues and adapting to new environments (7, 15,)The social withdrawal and stigma associated with NF1 may further exacerbate feelings of isolation and anxiety, as children with NF1 frequently encounter peer interaction challenges (15, 20).Moreover, somatization symptoms in NF1 children can be linked to increased health-related concerns and heightened sensitivity to physical symptoms, often exacerbated by their medical history and frequent clinical visits (20).

Some studies report that this is due to social isolation and medical uncertainty (20). Other studies suggest that the impact of COVID-19 on anxiety levels may vary depending on family environment, coping mechanisms, and access to psychological support systems (21). Children with NF1 are at higher risk for social difficulties due to deficits in executive function, emotional regulation, and peer relationships (21). Separation anxiety and social phobia are commonly reported, possibly due to difficulties in processing social cues and adapting to new environments (7, 15, 16). The social withdrawal and stigma associated with NF1 may further exacerbate feelings of isolation and anxiety, as children with NF1 frequently encounter challenges in peer interactions (15, 17).Moreover, somatization symptoms in children with NF1 may be linked to increased health-related concerns and heightened sensitivity to physical symptoms, often intensified by their medical history and frequent clinical visits (22).

## Conclusion

Our findings highlight the unique anxiety profile of children with NF1. The combination of neurobiological predisposition, environmental stressors, and social stigma underscores the importance of targeted psychological interventions for these children, particularly during periods of crisis. Early screening and tailored support are essential to mitigate emotional distress and promote better mental health outcomes in children with NF1.

## Data Availability

The datasets presented in this study can be found in online repositories. The names of the repository/repositories and accession number(s) can be found here: https://github.com/drcohenrony-png/Rony-Cohen.git.
